# T cell biomarkers come to the fore in cancer immunotherapy

**DOI:** 10.1016/j.xcrm.2023.100989

**Published:** 2023-03-30

**Authors:** Lozan Sheriff, Alastair Copland, David Bending

**Affiliations:** 1 Institute of Immunology and Immunotherapy, College of Medical and Dental Sciences, University of Birmingham, Birmingham, UK

## Abstract

A comprehensive study by van der Sluis et al.^1^ demonstrates immunotherapeutic targeting of OX40 and PD-L1 results in enhanced tumor clearance, which is linked to the dynamic emergence of distinct subsets of CD8^+^ T cells.

## Main text

PD-1 is upregulated upon activation of T cells and limits responses through attenuating T cell receptor^2^ (TCR) and CD28^3^ signaling. PD-1 and its ligand PD-L1 have proven to be attractive candidates for cancer immunotherapy, and their blockade has shown clinical efficacy in several cancer types with notable success when combined with anti-CTLA-4 therapy.^4^ However, despite these successes, most patients fail to respond to single-agent immunotherapy, and adverse event rates are frequently high.^4^ Therefore, there is an urgent need to rationally design more effective immune checkpoint therapy (ICT) combinations while at the same time seeking to limit potential side effects.

Van der Sluis et al.^1^ further our insight into effective ICT by undertaking a systemic analysis of T cell responses following an ICT combination targeting OX40 and PD-L1. OX40 is a co-stimulatory molecule that is part of the tumor necrosis factor receptor superfamily that has been previously identified as a potential target for cancer immunotherapy.^5^ OX40 is also a rational choice since multiple groups have demonstrated that T cells upregulate OX40 following anti-PD-1 treatment.^6^^,^^7^

The authors performed pre-clinical studies comparing the anti-tumor efficacy of single-agent PD-L1, an agonistic OX40 antibody co-delivered with CpG DNA, and the combination of PD-L1 and OX40/CpG DNA (henceforth referred to as PDOX). The PDOX approach was significantly more effective than all other regimes at slowing tumor growth in the MC38 and HCmel12 syngeneic cancer models. To explore the immunological consequences of PDOX treatment, they performed single-cell RNA sequencing (scRNA-seq) on blood circulating CD4^+^ and CD8^+^ T cells from tumor-bearing mice. Their analyses identified distinct CD4^+^ and CD8^+^ T cell subsets that were preferentially enriched in the circulation from mice that had received PDOX treatment. These T cells were marked by expression of the transcription factor *Id2* and the Galectin-1 gene, *Lgals1*, and expressed *Cxcr3,* a functionally important chemokine receptor. Strikingly the CD8^+^ T cell subset transcribed several NK cell-associated receptors, including *Klrk1* (encoding NKG2D), *Klrc1* (encoding NKG2A), and several genes encoding cytotoxic molecules (*Gzma*, *Gzmb*, and *Gzmk*). These data were confirmed at the protein level, including the intriguing finding that most CD8^+^ T cells from these clusters expressed a hyperglycosylated form of CD43.^8^ These results showed that PDOX treatment drove the emergence of CD8^+^ T cell subsets with heightened migratory and cytotoxic potential that were readily detectable in the blood.

Using an antibody to the hyperglycosylated form of CD43 (clone 1B11^8^), the authors were able to study the temporal dynamics of CD43-1B11^+^ T cells within the circulation of mice with MC38 tumors. These longitudinal studies showed that PDOX therapy led to an expansion in both CD4^+^ and CD8^+^ T cells that stained positive for CD43-1B11. Although not compared head-to-head directly, the effect of PDOX on these circulating T cell subsets appeared to be greater in mice with tumors than those without and much larger than in mice that had only received single-agent ICT. Stratification of mice by the frequency of CD8^+^ CD43-1B11^+^ T cells in the circulation showed a direct correlation of this subset with tumor clearance. Interestingly, systemic lymphoid tissue profiling suggested that there were interconnected changes in CD4^+^ and CD8^+^ T cell subsets, indicating that PDOX immunotherapy leads to system-wide changes in T cell subsets.

Importantly the authors could also identify similar T cell subsets within the blood of cancer patients receiving anti-PD-1 ICT. These included increased natural killer (NK) cell receptor expression on CD8^+^ T cells (KLRB1, KLRG1). Using publicly available data, they were able to demonstrate that NK cell receptor and cytotoxic gene signatures identified in mice also correlated with survival benefit in cancer patient cohorts.

The final part of the study sought to address mechanisms mediating tumor rejection in the PDOX setting by focusing on the T cell responses within the tumor microenvironment (TME). PDOX treatment drove a significant increase in the CD8^+^ T cell: Foxp3^+^ Treg ratio, including a proportional increase in CD4^+^ T helper cells. Given that Foxp3^+^ Treg cells are among the highest expressers of OX40, it is possible that PDOX treatment may also additionally benefit from the ability of OX40 antibodies to trigger Treg depletion in the TME, as has been reported in some experimental cancer models.^9^ Interestingly, using antibodies to deplete either CD8^+^ or CD4^+^ T cells, the authors clearly demonstrated that PDOX efficacy is mainly mediated by CD8^+^ effector T cells. Furthermore, the authors elegantly showed that the NK cell-associated receptor NKG2D and cell migration molecules CD43 and CXCR3 were all functionally important in mediating the anti-tumor effect of PDOX ICT in mice. Collectively, these experiments showed that many of the markers used to identify the unique T cell subsets in blood appeared to have functional importance in effecting the immune response to PDOX therapy.

With the rapid therapeutic effect of PDOX, it will be interesting to see how this ICT approach impacts different stages of T cell activation and exhaustion. The prevailing dogma within the field is that anti-PD-1 immunotherapy drives a proliferative burst in precursors to exhausted CD8^+^ T cells.^10^ Given this premise, is PDOX’s enhanced potency due to its ability to revive effector function across a wider subset of exhausted T cells? Or can PDOX broaden the T cell response to immunogenic tumors? Accordingly, it will be important to understand the precise role CpG DNA plays for enhancing the efficacy of OX40 ICT, since this adjuvant can interact with both APCs and T cells directly. Parsing of treatment components to uncover what is sufficient for therapeutic efficacy will greatly enhance our understanding of this exciting treatment strategy.

In conclusion, this study establishes clear evidence that circulating T cell signatures are a promising potential biomarker for monitoring responses to immunotherapy combinations, such as PDOX. Any future testing in clinical trials should consider longitudinal and systemic immune profiling to establish whether such “T cell biomarkers” can be informative in stratifying patient responses. In addition, it will be essential to test whether ICT combinations like PDOX can achieve increased efficacy while limiting adverse events.
